# Retraction: Acute psilocybin increased cortical activities in rats

**DOI:** 10.3389/fnins.2026.1787833

**Published:** 2026-02-04

**Authors:** Frontiers Editorial Office

The journal retracts the May 23 2023 article cited above.

Following publication, a reader contacted us to suggest that the compound used in this research may have been Psilocin-HCI rather than Psilocybin.

The authors confirmed that the compound used in this research was Psilocin-HCI. The conclusions reported in the article are no longer supported by the data. An investigation was conducted in accordance with Frontiers' policies that confirmed this; therefore, the article has been retracted.

The authors present updated findings in a new manuscript, Liu J, Wang Y, Xia K, Wu J, Zheng D, Cai A, Yan H and Su R (2026). Acute psilocin increased cortical activities in rats. *Front. Neurosci*. 20:1593703. doi: 10.3389/fnins.2026.1593703 (new submission: https://www.frontiersin.org/journals/neuroscience/articles/10.3389/fnins.2026.1593703/full).

This retraction was approved by the Chief Editors of Neuroscience and the Chief Executive Editor of Frontiers. The authors agree to this retraction.

Frontiers would like to thank the concerned reader who contacted us regarding the published article.

